# Marked to Die-Cell Death Mechanisms for Keratinocyte Acantholysis in Pemphigus Diseases

**DOI:** 10.3390/life12030329

**Published:** 2022-02-22

**Authors:** Valéria Bumiller-Bini Hoch, Larissa Schneider, Anna Elisabeth Pumpe, Emelie Lüders, Jennifer Elisabeth Hundt, Angelica Beate Winter Boldt

**Affiliations:** 1 Laboratory of Human Molecular Genetics, Department of Genetics, Federal University of Paraná, Curitiba 81531-980, Brazil; valeriabumiller@gmail.com (V.B.-B.H.); larischneider.01@gmail.com (L.S.); 2 Postgraduate Program in Genetics, Department of Genetics, Federal University of Paraná (UFPR), Curitiba 81531-980, Brazil; 3 Lübeck Institute of Experimental Dermatology, University of Lübeck, 23562 Lübeck, Germany; anna.pumpe@web.de (A.E.P.); ecl303@web.de (E.L.); jennifer.hundt@uni-luebeck.de (J.E.H.)

**Keywords:** pemphigus, cell death pathways, apoptosis, apoptolysis

## Abstract

Pemphigus is a group of blistering autoimmune diseases causing painful skin lesions, characterized by acantholysis and by the production of autoantibodies against, mainly, adhesion proteins. We reviewed the literature for molecules and/ or features involved in the 12 cell death pathways described by Nomenclature Committee on Cell Death, taking place in pemphigus patients, cell lines, or human skin organ cultures treated with sera or IgG from pemphigus patients or in pemphigus mouse models, and found 61 studies mentioning 97 molecules involved in cell death pathways. Among the molecules, most investigated were pleiotropic molecules such as TNF and CASP3, followed by FASL and CASP8, and then by FAS, BAX, BCL2, and TP53, all involved in more than one pathway but interpreted to function only within apoptosis. Most of these previous investigations focused only on apoptosis, but four recent studies, using TUNEL assays and/or electron microscopy, disqualified this pathway as a previous event of acantholysis. For PV, apoptolysis was suggested as a cell death mechanism based on pathogenic autoantibodies diversity, mitochondrial dysfunction, and p38 MAPK signaling. To answer those many questions that remain on cell death and pemphigus, we propose well-controlled, statistically relevant investigations on pemphigus and cell death pathways besides apoptosis, to overcome the challenges of understanding the etiopathology of pemphigus diseases.

## 1. Introduction

Pemphigus is an autoimmune blistering disease affecting the skin with painful and potentially lethal bullous lesions (reviewed by [1]). Based on the autoantibody reactivity, two major forms of pemphigus are differentiated. Pemphigus foliaceus (PF) is characterized by autoantibodies against desmoglein (DSG) 1, while DSG3 autoantibodies are characteristic of mucosal pemphigus vulgaris (PV), and reactivity against both autoantigens is seen in mucocutaneous PV [1]. Desmogleins are calcium-dependent adhesion molecules in the desmosomes that connect keratinocytes [2]. The binding of the autoantibodies to keratinocyte antigens leads to blister formation in the epidermis, caused by cell-cell detachment, a process called acantholysis, histologically hallmarked by intraepidermal split formation [1,3,4]. In PF, it takes place in the subcorneal layer of the epidermis but in PV, acantholysis occurs in the suprabasal layer [4]. In agreement with the DSG compensation hypothesis, the blistering sites differ between PF and PV due to the different expression sites of the DSG molecules in the epidermis: while DSG1 is mainly expressed in the upper layers, DSG3 is expressed in the lower layers of the epidermis [5]. However, the misconception of this hypothesis is that there are more desmosomal cadherins promoting the integrity of the epidermis, beyond the DSGs (reviewed by [6]).

The pathogenicity of IgG from pemphigus patients has been demonstrated through both in vivo and in vitro experiments [7,8,9]. However, the precise mechanism by which pemphigus autoantibodies induce blistering is not fully elucidated. One hypothesis to explain this is the direct inhibition of DSG transinteraction, where the binding of autoantibodies causes steric hindrance and therefore loss of cell adhesion, which leads to acantholysis [10,11,12,13]. However, this seems to be more applicable to PV than to PF [14]. Beyond steric hindrance, signaling pathways induced by PF or PV-IgG binding, such as phospholipase C [15], p38 mitogen-activated protein kinase (p38 MAPK) [16,17]; c-Myc [18], epidermal growth factor receptor (EGFR), Map kinase (ERK), transcription factor c-Jun [19] and phosphorylation of DSG3 [20], among others [21], have been proposed to trigger acantholysis by launching intracellular events that disrupt desmosome homeostasis. Indeed, inhibition of the MAPKp38 signaling pathways blocks keratinocyte dissociation [16,17,21].

Apart from DSG, a wide range of other proteins are targeted by pemphigus autoantibodies. Among these are adhesion molecules, cell membrane receptors, and mitochondrial antigens [6,22]. These complex pathological mechanisms hinder the development of new treatment options, which remain based on oral corticosteroids [23], besides intravenous immunoglobulins (IVIg) [1,24]. Although the mechanistic reasons for IVIg treatment success are not fully understood, it protects target cells from apoptosis by interfering with signaling pathways, increasing the sensitivity to corticosteroids [24]. Recently, the anti-CD20 antibody rituximab, combined with corticosteroids, was found to induce complete remission off-therapy in almost 90% of patients [1,25].

Pemphigus occurs sporadically around the world. PF incidence is 0.75–5 cases/million per year [26] and PV incidence has a range to 0.6 in Switzerland to 32 in Israel (reviewed by [1]). Despite its worldwide sparing distribution, PF is endemic in South America and Tunisia [27,28,29,30]. In Brazil, endemic PF (EPF) is commonly known as fogo selvagem, “wild fire” in Portuguese, due to a popular belief that the blisters and burning sensation resulted from a curse or tribal rituals [31]. It is called “El Bagre” in Colombia, named after the gold-mining endemic region [32]. Endemic PF in Brazil reported the highest prevalence in 1996 (3.04%), in Limão Verde (Mato Grosso do Sul) [33].

Pemphigus is a multifactorial disease, resulting from a combination of genetic, epigenetic, and environmental factors [1,23,34,35,36]. The strongest genetic risk signature of pemphigus disease is the association with HLA class II alleles in several populations around the world (reviewed by [36]). Besides that, alleles from genes involved in many other mechanisms of the innate and adaptive immune system, including cell death pathways, were associated with pemphigus (reviewed by [36]); [37].

Among Brazilian PF environmental factors are bites of the sand (Phlebotominae) and black (Simuliidae) flies [38,39]. It is hypothesized that salivary antigens from these flies induce a cross-reaction leading to autoantibody production against DSG1 [40]. However, those flies also occur in Northern regions, with much lower, although growing, the incidence of the disease [41]. The possibility of a virus or other microorganism as triggers of the disease was also suggested [30,31]. Furthermore, the epidemiological landscape seems to follow the deforestation border, creating geographical clusters [39]. It was also observed that thiol, other calcium-sequestering components [31], and UVB radiation due to sunlight exposure might have an impact on the susceptibility of PF [42]. Epidemiological differences are not followed by any histopathological features that would distinguish endemic, from non-endemic PF. They are identical [43], except for higher anti-DSG1 IgM and IgE serum levels in endemic PF [44,45,46]. In contrast to PV, association with other autoimmune diseases and longitudinal mother-child transmission are very rare [30,47,48,49,50,51,52,53,54]. If affected, newborns soon recover [47]. Ruocco et al., 2013 listed several environmental factors associated with PV, such as thiol and phenol drugs, previous viral infections, sunburns, ultraviolet and ionizing radiation, surgical and cosmetic procedures, dietary factors, and emotional stress, all of them supposed to facilitate the development of pemphigus [34]. Yet, low levels of 25-hydroxyvitamin D (25OHD) were reported in the serum of 34 PV patients compared to the healthy controls [55].

Here, we reviewed the connections between pemphigus and cell death, an issue that has been repeatedly studied (reviewed by [56,57,58]). Cell death is an essential mechanism to tissue homeostasis and protection against diseases such as cancer, infection, and autoimmune disorders [59]. According to the Nomenclature Committee on Cell Death (NCCD), there are 12 pathways orchestrating the cell death [60]. In a recent genetic association study of our group with EPF, we identified an association with polymorphisms of genes directly involved in apoptosis resistance, as well as gene variants whose products induce and regulate pyroptosis, necroptosis, necrosis, parthanatos, and immunogenic cell death pathways [37]. To our knowledge, only apoptosis had been investigated before in pemphigus [17,61,62,63,64,65,66], and some of the studies disqualified apoptosis as the major cell death mechanism [17,63,64,66].

Apoptosis and necrosis are the best-known ways of cell death. Apoptosis is a regulated cell death pathway that does not entail the activation of inflammation [67]. There are two ways of activating apoptosis by cysteine aspartate proteases, called caspases. Intrinsic apoptosis is the result of cell damage like DNA degeneration and extrinsic apoptosis is mediated by signaling molecules like TNF (reviewed by [60]). Caspases can also be activated independently of apoptotic signaling (reviewed by [68]) and cleave several adhesion molecules like DSG [69,70]. Apoptosis leads to the formation of apoptotic bodies that are removed by phagocytic cells (reviewed by [60]). In contrast, necrosis is the uncontrolled response of cells to tissue damage that results in cell death. The dying cells release their cell content into the environment so that necrosis is followed by an immune response (Reviewed by [71]).

**Figure 1:**
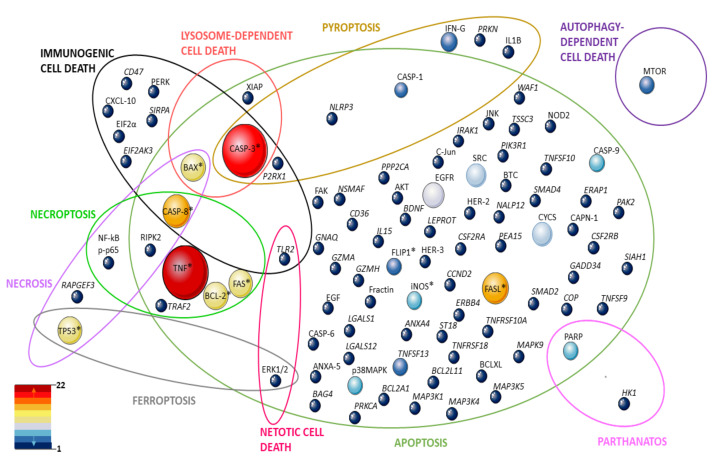
Representativeness of molecules in studies on cell death mechanisms and pemphigus. Ellipses represent cell death pathways and the intersections between them. The molecules investigated at the genetic, expression, and protein levels are shown by circles whose size corresponds to the number of articles in which they were investigated. They are distributed according to the pathways they are part of. The colors reinforce the representation of these molecules in the reviewed articles (dark red–most investigated, dark blue poorly represented). * molecule investigated both at the gene and protein level.

Until some years ago, cell death pathways were summarized as apoptosis and necrosis. In this review, we aimed to open new views for the investigation in pemphigus considering other cell death pathways described by NCCD [60]. These include reactions of cells to different stress situations like overproduction of ROS (ferroptosis) or the accumulation of different proteins (parthanatos) or infection with a pathogen (necroptosis, pyroptosis). They differ from each other by the way they activate or regulate inflammation (passive or active) (reviewed by [72]). We laboriously reviewed the literature for molecules and/or features involved in cell death pathways described by NCCD, taking place in the pemphigus patient, cell lines or human skin organ cultures (HSOC) treated with sera or IgG from pemphigus patients, or pemphigus mouse models, providing an updated overview of this subject and perspectives for the future (the strategy search is described at the Supplementary Materials, Supplementary Figure S1). We compiled 61 studies for this review (Table 1), where 97 molecules, to the best of our knowledge, involved in cell death pathways were investigated in pemphigus (Figure 1).

## 2. Results and Discussion

### 2.1. Signaling Pathways: TNF, FAS, and Other Proteins

We found nine studies that investigated the association of pemphigus with polymorphisms of genes whose products are involved in cell death pathways (Supplementary Table S1). One of them investigated the association of polymorphisms of genes of all 12 cell death pathways (intrinsic apoptosis, extrinsic apoptosis, mitochondrial permeability transition (MPT)-driven necrosis, necroptosis, ferroptosis, pyroptosis, parthanatos, entotic, NETotic, lysosome-dependent, autophagy-dependent and immunogenic pathways, listed by the NCCD [60] in a case-control study of Brazilian EPF. There was association evidence for genes whose products are directly involved in apoptosis (TNF rs1800630*A, TRAF2 rs10781522*G, CD36 rs4112274*T, PAK2 rs9325377*A), as well as that induce and regulate pyroptosis (PRKN rs9355950*C), necroptosis (TNF rs1800630*A, TRAF2 rs10781522*G), necrosis (RAPGEF3 rs10747521*A), parthanatos (HK1 rs7072268*T), and immunogenic cell death (EIF2AK3 rs10167879*T, SIRPA rs6075340*A, CD47_rs12695175_G) pathways. This last pathway can be activated by environmental factors associated with PF susceptibility, such as UV irradiation, calcium-sequestering components, and components of fly saliva [37]. In a genome-wide association study with three PV populations, a variant rs23043658*A at gen*e* C2H2C-type zinc finger transcription factor (*ST18*) which encodes a pro-apoptotic molecule, was associated in Jew and Egyptians. Also, its expression was upregulated in 8 skin biopsies of PV patients [112]. Seven of the association studies, done with PF and/or PV, focused on genetic polymorphisms of tumor necrosis factor (*TNF*) [37,73,86,91,101,111,119] (Supplementary Table S1). TNF-alpha is a proinflammatory cytokine involved in both apoptosis and necroptosis, mainly associated with the severity of different immune-regulated diseases [123], such as pemphigus [124]. Its encoding gene is located in 6p21.33 in the Major Histocompatibility Complex (MHC) class III region and is produced by macrophages, monocytes, neutrophils, T cells, and NK cells [125]. TNF-alpha binds to TNFR1 and TNFR2 receptors and ultimately regulates cell proliferation and differentiation, and the immune response [126]. Three polymorphisms may modulate the susceptibility to PF: rs1800630, rs1800629, and rs361525. TNF rs1800630*A, associated with lower *TNF* gene expression and protein levels [127], occurs in absolute linkage disequilibrium with the major rs1800629*G and rs361525*G alleles and was associated with the Brazilian EPF susceptibility [37]. Both the minor rs361525*A allele and the rs1800629*G_rs361525*A (*GA*) haplotype were associated with protection against the Brazilian EPF [111]. This was expected, since in contrast with the rs1800630*A allele, the rs361525*A variant is associated with a higher *TNF* gene expression [128,129]. The rs1800629*GA+AA was associated with a higher risk of sporadic pemphigus disease in Egypt for PV [101] and rs1800629*A in a Polish population with PF [119]. In contrast, the most common rs1800629*G_rs361525*G (*GG*) haplotype was associated with PV susceptibility in the Slovak population [91]. It was not associated in a small Argentinian study of 20 pemphigus patients [86]. *TNF* microsatellite loci (STR)*2 and *5 were associated with EPF susceptibility in Tunisia, while TNF*6 with protection. Importantly, the susceptibility alleles are not in significant linkage disequilibrium with the main alleles associated with PF worldwide: DRB1*04, as well as the most associated in the Tunisian population, DRB1*03 [73]. 

Higher TNF expression has been repeatedly associated with PV and PF [81,83,92,108] (Table 2 and Supplementary Table S2). However, TNF serum levels did not differ between 20 PV patients [117], as well as in 19 PV patients in the active stage and 24 patients in clinical remission compared to controls [102]. TNF serum levels were positively correlated with autoantibody titers, disease duration, and lesion numbers in the PV patients [74]. They were also higher in 25 PV Iranian, 10 PV Egyptian, and 25 PV Italian patients and a PV Japanese case report [75,92,108]. In the Italian PV patients, TNF and interleukin 6 (IL-6) levels were high at first and decreased after one month of therapy [83]. In agreement, *TNF* gene expression was evident in the epidermis of 19 PV patients [88]. This occurs especially near the blister, while it was almost not found in nonlesional skin of 6/7 PF and 5/6 PV patients [97]. TNF protein was also increased in the lesional and perilesional epidermis in 2/5 PV patients and lesional and perilesional dermis of all PV patients [80]. In addition, TNF was detected in the inflammatory exudate of more than 70% of 13 investigated EPF patients [65] and in the supernatants from peripheral blood molecular cells (PBMC) cultivated from 12 Brazilian EPF and 7 PV patients [81]. Pretreatment with anti-TNF strongly inhibited complement *C3* gene expression and about 80% of cell detachment in normal human epidermal keratinocytes (NHuK) incubated with PV-IgG (IgG immunoglobulin obtained from PV patients). With the addition of anti-IL-1α, acantholysis inhibition reached 84% [87,88]. Higher levels of IL-1B were observed in supernatants from Brazilian EPF PBMC even during the glucocorticosteroid therapy [110]. A lower incidence of PV-similar blisters also occurred in TNF receptor knockout mice (*TNFR1R2-/-*) after PV patient serum injection, compared with control wild-type mice (54 vs. 87%, *p* < 0.05) ([88]). Chiapa-Labastida [80] suggested that the accumulation of apoptotic cells in PV patients might enhance TNF production, which in turn activates apoptosis in more keratinocytes. 

**Figure 2:**
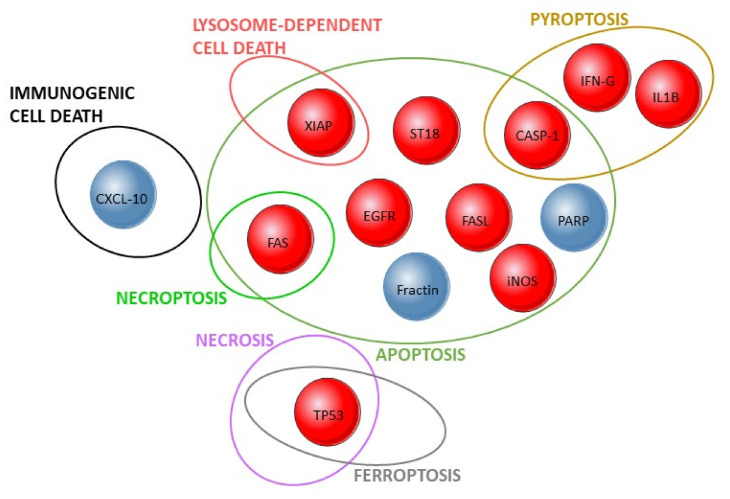
Validated results in human studies on cell death mechanisms and pemphigus. Ellipses represent cell death pathways and the intersections between them. The investigated proteins are shown by circles whose red color corresponds to their detection or increased expression in pemphigus patients, whereas blue corresponds to absent, low, or decreased expression. Discrepant results in the literature were excluded.

Another relevant and highly investigated protein in pemphigus is Fas [24,61,65,100,105,107,113,120] (Figure 2, Table 2 and Supplementary Table S2). The Fas protein is a membrane receptor of the TNF receptor family. Upon binding with Fas ligand (FasL), it triggers apoptosis, having fundamental importance in the maturation process of T and B lymphocytes and the homeostasis of the immune system [130]. Thus, mutations or unwanted activation of Fas may have very negative repercussions. Epidermal PV lesions expressed Fas and FasL proteins, as well as perilesional PV skin for Fas [61,105,120]. Fas was also expressed on keratinocytes of the lesional epidermis of 2/5 PV patients [113], and in the epithelium and the cells of the inflammatory exudate of 3 and 10 of 13 EPF patients, respectively [65]. Furthermore, NHuK treated with PV-IgG from two different PV patients presented higher *FASL* gene and FasL expression. After IVIg treatment, the PV-IgG of these patients did not induce *FASL* expression [24]. Interestingly, NHuK and HSOC treated with PV-IgG and/or FasL and/or TNF presented increased cell death and acantholysis [104]. FasL levels also increased in the supernatant of cultivated NHuK and HSOC treated/injected with PV-IgG, respectively. Moreover, the perilesional area of NHuK cultivated with PV-IgG presented enhanced expression of both Fas and FasL. Soluble FasL (sFasL) levels increased before and after blister formation, but membrane FasL numbers increased after blister formation [120]. Patients with mucosal PV had the highest mean value of sFasL (2775 pg/mL-2 patients), followed by the mucocutaneous group (1820 pg/mL-5 patients) and patients with only cutaneous involvement (1130 pg/mL-3 patients) [100]. In addition, sera from untreated pemphigus patients presented higher FasL levels than sera from corticosteroid treated patients (23 PV and 13 PF patients) and induced NHuK apoptosis [107]. FasL may induce keratinocyte apoptosis through caspase (CASP)-8 activation, based on the evidence that (1) FasL expression increased, followed by CASP-8 activation and by TUNEL-positive epidermal cells in NHuK treated with PV-IgG before acantholysis [61], (2) anti-FasL or CASP-8 inhibitor decreases cell death [107] and (3) FasL silencing through siRNA reduces PV-IgG-induced CASP-8 activation and Dsg3 cleavage in NHuK treated with patient IgG [61]. In a mouse model, anti-FasL injection greatly reduces blister formation after PV-IgG injection. Mutant mice without sFasL also had reduced acantholytic area, whereas mice with sFas, but lacking the membrane form of FasL, presented blisters after PV-IgG injection [61]. Interestingly, NHuK and HSOC treated with PV-IgG and/or Fas-L and TNF presented increased cell death and acantholysis [104]. Finally, serum levels of the apoptosis inhibitors livin and X-linked inhibitor of apoptotic proteins (XIAP) increased in all seven investigated pemphigus patients (5 PV and 2 PF) after IVIg treatment [118]. Interestingly, pathogenic anti-Dsg3 antibodies activate apoptosis in a FasL-independent way in cultured keratinocytes, increasing combined Annexin V and propidium iodide (PI) positivity at 4 h, with a peak at 12 h post-treatment. This leads to the “2-hit hypothesis”, where the first hit of both pathogenic and non-pathogenic antibodies cause structural cell membrane changes (cell stiffness), and the second hit is only mediated by pathogenic antibodies and induces FasL-independent apoptotic processes [114].

PV perilesional skin also expresses EGFR ([19]). This receptor may induce apoptosis if overexpressed [131]. PV-IgG injection did not change *EGFR* expression but increased the levels of autophosphorylated EGFR in NHuK and HSOC, 30 min after PV-IgG treatment. The numbers of phosphorylated extracellular signal-regulated kinases ERK1/2 did also increase and were detectable after 15 min, lasting up to 10 h. The plausible substrate of ERK, c-Jun, was phosphorylated after 4 h of treatment. Treatment with EGFR inhibitor (AG1478) or MAP (mitogen-activated protein) kinase (ERK) inhibitor (PD98059) reduced the numbers of apoptotic cells and CASP-3 activity in NHuK incubated with PV-IgG. The EGFR inhibitor also reduced Fas-L levels, whereas the MAPk inhibitor reduced the numbers of phosphorylated ERK1/2 and c-Jun. In conclusion, PV-IgG binding to membrane receptors activates EGFR and leads to dissociation of plakoglobin (cytoplasmic desmosomal component), phosphorylation of ERK and c-Jun, followed by an increase in sFasL secretion and organization of death-induced signaling complexes (DISC, composed by Fas, FADD, or Fas-associated protein with death domain and CASP-8) [19].

Global peripheral blood gene expression in PV patients and controls revealed many differentially expressed genes. A highlighted functional pathway was apoptosis (Supplementary Table S2). Several pro-apoptotic genes were upregulated in patients with active clinical PV [85]. Genome-wide gene expression profiles of peripheral CD4+ T cells comparing (1) Brazilian EPF patients vs. controls, (2) Brazilian patients with generalized vs. localized forms of EPF, and (3) untreated vs. treated Brazilian EPF patients, revealed many differentially expressed genes of programmed cell death (GO:0012501) (Supplementary Table S2) [98]. Among the genes encoding anti-apoptotic proteins and with decreased expression in T lymphocytes of patients undergoing corticosteroid treatment, *BCL2A1* (B-cell lymphoma 2-related protein A) stands out. Its product sequesters pro-apoptotic proteins B-cell lymphoma 2 (BCL) family, reduces the release of pro-apoptotic cytochrome c (CytC) from mitochondria, and inhibits caspase activation, promoting lymphocyte activation and survival [132]. Eleven and 4 of 13 Brazilian EPF patients expressed the antiapoptotic BCL-2 protein in the cells of the inflammatory exudate and the epithelium, respectively [65]. In agreement, NHuK or HSOC reduced BCL2 apoptosis regulator (*BCL2*) gene and protein expression after incubation with PV serum or PV-IgG [76,120]. BCL-2 plays a role in maintaining epithelial cell integrity and may inhibit the Th17 activation [133]. The Th1/Th17 immune responses play an important role in the pathogenesis of PV, with increased proapoptotic interferon-gamma (IFN-gamma) and reduced chemokine interferon gamma-induced protein 10 (CXCL-10) levels in 20 investigated PV patients [117]. Most of the 13 investigated EPF patients mentioned before (11/13) also presented proinflammatory inducible nitric oxide synthase (iNOS), interleukin 1 (IL-1), and IFNg in the inflammatory exudate [65]. NHuK incubated with PV serum also overexpressed *iNOS* and its product [76] creating high levels of reactive oxygen species (ROS). NHuK cultivated with sera from PV patients also increased levels of phosphorylated (p) pPERK (RNA-dependent protein kinase (PKR)-like ER kinase) and pEIF2α, a translation initiation factor that acts as a central hub of the stress response [94]. PERK is required at the contact sites between the endoplasmic reticulum (ER) and mitochondria to elicit apoptosis after ER stress-mediated by ROS [134].

Nguyen et al. looked into the antagonizing effects of PV-IgG and methylprednisolone (MP) in human keratinocytes on a genomic level. Their results showed that PV-IgG leads to a decrease in the transcription of 198 different genes and an enhanced transcription of 31 genes, whereas some of these genes are regulated reciprocally by MP. In keratinocytes treated with PV-IgG, the transcription of apoptosis-related genes like p53-activated fragment-1 (*WAFI*), IPL protein (*TSSC3*), apoptosis-associated protein (*GADD34*), phosphoprotein PEA-15, and cyclin D2 was down-regulated compared to the controls (Supplementary Table S3). On the other hand, the gene encoding proapoptotic *BAX-δ* is up-regulated, when comparing MP + PV-IgG vs. PV-IgG [103].

PV lesional skin also expressed the pro-apoptotic Bcl-2-associated X protein (BAX), as well as its regulator, tumor suppressor protein Tp53 [120] (Supplementary Table S4). BAX expression seemed at first unimportant in PV patients [84] but was later found increased in NHuK cultivated with PV sera [109] (Supplementary Table S4). The same treatment increased the *TP53* gene and protein expression [76,109]. In accordance, TP53 expression was higher in 48% of lesional and non-lesional skin biopsies of 25 PV patients. This seems to be a direct consequence of desmosomal disruption since intact DSG3 was found to inhibit TP53 expression and activity [109]. However, susceptibility to Brazilian EPF was not associated with two common polymorphisms of the corresponding genes, namely *BAX -248 G > A* (may alter RNA splicing, rs4645878) and *TP53 12139 G > C* (p.Arg72Pro, altering structure and function of the mature protein, rs1042522) [93]. The expression of the NLR family pyrin domain containing 3 (*NLRP3*) gene, whose product is a central player in the innate immunity [135], did not differ in 43 oral PV patients compared with 40 health controls [115].

Apoptosis progression is regulated in an orderly way by a series of signal cascades, coordinated by 14 proteases of the interleukin-1β-converting enzyme family that play important roles in inflammation (caspases-1, -4, -5, and -11), in the cell cycle (CASP-2), in cell differentiation (CASP-14) and apoptosis. The last function divides these molecules into initiator (caspases-8, -9 and -10) and effector (caspases-3, -6 and -7) caspases [136]. Acantholytic cells of 17 PV biopsies presented *CASP3* gene and protein expression [105], and both lesional and perilesional epidermis and dermis from PV patients presented an increased number of cells with activated CASP-3 [80,109]. Higher CASP-1 and/ or CASP-3 expression was detected in NhuK and HSOC treated/ injected with PV-IgG [19,24,118,119], although the opposite for CASP-3 was true in another study [68]. After treatment of NhuK with PV-IgG, activated CASP-3 increased concomitantly with cell death (evaluated by methylene blue) and acantholysis [89].

As already mentioned before, CASP-8 is activated by the binding of FasL to the Fas receptor in the apoptosis extrinsic pathway. Although apoptosis was proposed to precede acantholytic detachment because CASP-8 activation preceded disruption of cell-cell contacts, this issue seems far from being resolved. Activated CASP-8 was detected in PV skin and higher CASP-8 levels were observed after [120] and before [89] blister formation in NhuK treated with PV-IgG. Interestingly, CASP-1 inhibitor (YVAD-CHO) decreased cell death and acantholysis in NhuK cultivated with PV-IgG (evaluated by trypan blue dye–TBD staining) and decreased acantholysis in HSOC injected with PV-IgG [120]. In accordance, higher *CASP3* and *CASP8* gene and protein expression were observed in NhuK treated with PV-IgG from two patients. After IVIg treatment, the PV-IgG lost its association with the expression of these genes, as well as with *FASL*, induced higher expression of the anti-apoptotic FLIP-I (FLICE-inhibitory protein) and reduced cell detachment and numbers of cells with double-strand DNA breaks (detected with the TUNEL assay terminal deoxynucleotidyl transferase-mediated dUTP nick end labeling) positive cells. Similar consequences were observed in the murine epidermis [24].

Apoptotic cells attract mononuclear phagocytes like CD1a+ and CD14+. CD1a+ phagocyte numbers increased not only in the perilesional epidermis but also in the lesional and perilesional dermis of 5 active untreated PV patients. In the lesional and perilesional dermis, CD14+ cells also appeared in higher numbers. The authors of this study hypothesized that the mononuclear phagocytes may not easily reach the epidermis due to the high number of apoptotic cells in the dermis, which may explain their absence in the lesional epidermis [80]. 

### 2.2. Cell Death Controversy

#### 2.2.1. TUNEL Assays–Are the Cells Dead or Alive?

The TUNEL assay allows the identification of endonuclease-driven DNA fragmentation (causing 3′-OH DNA termini), associated with different mechanisms of cell death, including but not restricted to apoptosis [137]. TUNEL+ cells were reported in 17 PV biopsies [105], in perilesional biopsies of 23 PV and 13 PF patients [107], in the lesional and perilesional epidermis and dermis of 5 PV patients [80], as well as in the epithelium (12/13) and inflammatory exudate (11/13) in Brazilian EPF patients [65] (Table 3). The PV blister roof was positive, lending support to the hypothesis that cell death precedes acantholysis [120]. Biopsies of 4 PF and 4 PV were positive for multiple pycnotics and condensed nuclei stained with TUNEL, extending beyond the region of acantholysis, even reaching the normal epidermis. PF biopsies presented abundant TUNEL+ cells clustered in the subcorneal zone and only single TUNEL+ keratinocytes in the upper spinous layer where the epidermis was cohesive. This may suggest an early onset of cell death in pemphigus, meaning that DNA fragmentation occurs before the development of frank acantholysis [62]. In 72% of 25 PV mucosa biopsies, the frequency of TUNEL+ cells was higher than 75% in the basal layer of perilesional tissue, while it was 100% in the tombstone region (*p* = 0.038). TUNEL positivity was also abundant in the parabasal region and the roof of the vesicles. Positivity was higher in the basal than in the parabasal layer, as in the tombstone area compared to the roof of the blister (*p* = 0.0005 and *p* = 0.038, respectively) [84]. In agreement, 14 of 15 PV patients showed TUNEL positivity in the basal layer, 13 in the blister roof, and 12 presented TUNEL+ acantholytic cells. Twelve of 15 controls presented TUNEL+ cells, but only in the granular layer [82]. NhuK incubated with PV serum or PV-IgG also presented TUNEL+ cells [76], as well as TBD+ cells [24] with TBD+ lesion edges in not detached cells and in perilesional area, indicating cell death [120]. TBD staining also indicated cell death in NhuK and HSOC treated with PV-IgG and/or Fas-L and TNF, along with increased acantholysis [104]. Cell viability, measured by TBD, decreased to 10% in HSOC treated with plasma from PV, while the viability was 60% in the controls [116]. In agreement, after acantholysis, TBD vital staining and annexin V-Cy3 showed dead cells at lesional and non-lesional areas in NhuK treated with PV-IgG. In HSOC injected with PV-IgG, through MTT (3-[4,5-dimethylthiazol-2-yl]-2,5-diphenyl tetrazolium bromide) assay, cell viability decreased with increasing time of exposure and PV-IgG concentration [19]. In a PF mouse model, TUNEL+ cells were detected 8 h after PF IgG injection, whereas initial histological blisters were revealed after 12 h PF IgG injection. In addition, up-regulation of the expression of (pro-apoptotic) Bax at 2 and 4 h after PF IgG injection was reported [95], detection of cleaved CASP-3 and activated CASP-6 after PF IgG injection [95], as well as of da down-regulated expression of (anti-apoptotic) Bcl-xl at 6, 8 and 20 h after PF IgG injection [95]. Lastly, the administration of caspase inhibitors blocked PF blistering, and TUNEL was negative [95] (Supplementary Table S5).

In contrast, 10 Brazilian EPF showed no difference regarding TUNEL detection between injured PF skin and healthy skin. Few cells were positive in both groups. On the other hand, a stem marker protein, P63, was highly expressed in both tissues [122]. Schmidt et al. [64] did not detect hallmarks of apoptosis in perilesional skin sections from two PV patients. TUNEL+ cells were found solely in the blister roof or around the intraepidermal split from 2 of 4 biopsies of one PV patient. In the other PV patient, in areas with intraepidermal splits, 1 of 4 biopsies presented TUNEL+ cells [64]. Positive cells for cleaved caspases could not be observed. HaCaT and NhuK cells were incubated with PV-IgG from 5 PV patients but no apoptotic characteristics were visible. They neither observed changes in the nuclear morphology nor increased TUNEL positivity although acantholysis was induced (visible through fragmented Dsg 3 immunostaining, cytokeratin retraction, and cell dissociation). Inhibition of caspases (z-VAD-fmk) or overexpression of FLIP did not prevent acantholysis in cultured cells, supporting the conclusion that apoptosis is not a necessary condition for PV acantholysis [64]. In agreement, Janse et al. found few TUNEL+ cells in 11 mucocutaneous PV biopsies (3 lesional, 3 perilesional, and 5 healthy skin), and 11 PF biopsies (5 lesional, 2 perilesional, and 4 healthy skin). They found no CASP-3, CASP-8, fractin and nuclear PARP (Poly [ADP-ribose] polymerase) in the biopsies, as well as in PV and PF HSOC models [63]. Accordingly, human HaCaT keratinocytes and epidermis of adult and neonatal mice presented no fragmented nuclei after PV-IgG treatment, neither during the formation of the lesion nor after acantholysis. Similar to Janse’s study, the analysis of six skin biopsies from PV patients didn’t show TUNEL positive cells in non-lesional, perilesional, or most lesional tissue [68]. Moreover, maximal 7.5% and 3.5% of the cells in HSOCs injected with PV-IgG or with PF-IgG, respectively, were TUNEL+, depending on the extent of incubation time. There was no difference between healthy and lesioned skin Fields [63].

#### 2.2.2. Caspase Activity—Are the Cells Apoptotic or Not?

Apoptosis requires that CASP-3 and other caspases undertake poly ADP-ribose polymerase (PARP) cleavage [138]. However, neither a global actin collapse nor reduced full-length PARP nor increased cleaved PARP was observed in human and mouse keratinocytes and mouse epidermis after treatment with an experimental Dsg3 monospecific antibody (AK23) or PV-IgG [68], in agreement with the results obtained by others with 11 mucocutaneous PV and 11 PF biopsies [63]. Instead, there was an early transient, low-level activation of CASP-3 in mouse keratinocytes and AK23-treated adult mice one hour after AK23 or PV-IgG treatment, just before blistering. Furthermore, CASP-3 inhibition reduced acantholysis in cultured keratinocytes, neonatal and adult mice but inhibiting other caspases failed to prevent loss of cell adhesion. Thus, CASP-3 activation seems independent of the apoptotic signaling cascade, and apoptosis itself seems not needed for acantholysis in these PV models [68].

### 2.3. Morphology

Wang et al. (2004) investigated the different effects of PV-IgG on young (1–2 passages) and old (4–5 passages) cultured keratinocytes. Detachment of the cells occurred to a greater extent in aged cells as well as the number of dead cells whose death was induced with PV-IgG [121]. Higher FasL (m- and s-FasL) concentrations, DNA fragmentation, blebbing membranes, and apoptotic bodies confirmed these results. Activities of CASP-1, -3, and -8 also increased in aged cells, being consistent with down-regulated BCL-2 and increased p53. All in all, PV-IgG-induced apoptosis was evident in senescent cells, which might explain the higher severity and frequency of PV in older people [121]. Apoptotic cells (degraded cells and nuclei with condensed chromatin) were also reported in histological sections of lesional PV patients’ skin stained by hematoxylin and eosin [89].

However, using electron microscopy, considered the gold standard for the identification of apoptotic cells [63,139]), no apoptotic features were observed in acantholytic, lesional PV, and PF skin [63]. In agreement, no nuclear changes were observed in perilesional skin biopsies of 2 PV patients and HaCaT culture incubated with PV-IgG [64].

### 2.4. Apoptolysis and Mitochondria

Grando et al. (2009) proposed that apoptosis followed acantholysis in the apoptolysis pathway, by the means of: (1) binding of three classes of autoantibodies against desmosomal autoantigens, mitochondrial autoantigens as acetylcholine receptors, and other autoantigens on the plasma membrane of keratinocytes, acting synergistically with proapoptotic serum and tissue factors; (2) activation of EGF receptor, Src, mTOR, p38 MAPK, and other signaling elements downstream of ligated antigens, elevating intracellular calcium levels and launching cell death cascades; (3) basal cell shrinkage by caspases that disrupt cytoskeleton components and then phosphorylate and cleave the cytoplasmic tails of transmembrane cadherins, dissociating inter desmosomal adhesion complexes; (4) massive collapse of structural proteins and production of scavenging antibodies, accompanied by total desmosome separation; (5) apoptotic death of acantholytic cells resulting from irreversible damage to mitochondrial and nuclear proteins [140].

In agreement with mitochondrial injury, PV sera disrupted the electron transfer chain and the electrochemical gradient of KCs, increasing proton leakage and the production of reactive oxygen species (ROS). The mRNA expression of *BAX, CASP3*, nucleotide-binding oligomerization domain containing 2 (*NOD2*), receptor-interacting serine/threonine kinase 2 (*RIPK2*) and nuclear factor kappa B p-p65 (*NFkB p65*) increased and of *BCL2* decreased in human KCs treated with PV serum (Supplementary Table S3). These effects, as well as ROS production, stimulated either by UVB or PV serum, reversed by antioxidant naringenin (pre)treatment, preventing cell death [96]. Furthermore, PV patients produce PV-IgG against different mitochondrial proteins and in different amounts [22,78,99]. The absorption of antimitochondrial antibodies blocked/reduced the capacity of PVIgG to induce acantholysis in KCs and a PV mouse model, respectively. Pretreatment with a combination of all three mitochondria-protecting drugs minocycline, nicotinamide, and cyclosporine A almost completely abolished acantholysis in the mouse skin [22]. Specifically, the serum of five PV patients contained antibodies targeting subunits of mitochondrial nicotinic acetylcholine receptors (mt-nAChRs), which normally keep the mitochondrial permeability transition pore (mPTP) closed. Stimulation of these receptors prevented the release of CytC through the mPTP and therefore the formation of the apoptosome [78]. Another three non-desmoglein antibodies causing skin acantholysis after injection in neonatal mice were identified in the sera of 12 PV patients lacking antibodies against desmoglein 1 or 3. They targeted desmocollin 3 (DSC3), the muscarinic acetylcholine receptor subtype M3 (M3AR), or the secretory pathway calcium ATPase (SPCA1), leading to higher CytC release. Adsorption of these autoantibodies prevented blister formation and a positive Nikolskiy sign in mice. Furthermore, the effect of these autoantibodies on apoptolysis markers was studied in cultured human keratinocytes. All of the three autoantibodies lead to an increase in the activity of p38 MAPK and CASP-9, important steps in apoptolysis signaling. Anti-Dsc3 and anti-SPCA1 also activated the Src kinase. Inhibition of SPCA1 through the autoantibodies triggered apoptotic signaling due to accumulation of Ca2+ and Mn2+ [79].

PV-IgG binds to neonatal Fc Receptor (FcRn) for internalization and intracellular trafficking of PVIgG to mitochondria. Among these PVIg, anti-mitochondrial antibodies (AMA) and anti-Dsg synergize to cause acantholysis in the mouse model, increasing CytC release and changing the membrane potential of mitochondria, which might trigger the intrinsic apoptotic pathway. All these processes and the shrinkage of KCs were abolished by blocking FcRn [77]. PV-IgG binding leads to the activation of EGFR, tyrosine kinase Src, p38 MAPK, and c-Jun N-terminal kinase JNK in KCs with different time patterns, according to each patient. Patient source of PV-IgG further seems to influence: (1) the number of TBD-positive cells in cell monolayers, (2) acantholytic activity in a mouse model, (3) the response to CASP-3 inhibitors (DEVD-CHO, Z-DEVD-FMK, and Z-DCB-MK) and calpain inhibitors (MDL-28170, PD-150606, and CSP) in cell culture. Thus, PV-IgG from different PV patients seems to induce acantholysis through cell shrinkage (apoptosis) or cell swelling (oncosis preceding necrosis) [24]. Inhibitors of executioner caspases did not change EGFR, Src, and the first peak of p38 MAPK, but decreased JNK, the second peak of p38 MAPK, and CytC in keratinocytes treated with PV-IgG [99] (Supplementary Table S4).

Thus, irreversible mitochondrial damage is probably the reason for elevated CytC in keratinocytes treated with PV sera or pure PV-IgG fractions [77,99]. Elevated CytC, CASP-3, 8, and 9 confirmed activation of the intrinsic and extrinsic apoptosis pathways in keratinocytes treated with PV sera or pure PVIgG fractions, being PV sera much more effective in activating FasL and subsequently CASP-3 and 8. Anti-FasL decreased CASP-8 activation in both groups, and CASP-3 in KCs treated with PV-sera. However, the addition of anti-FasL antibodies did not modify CASP-9 activity and CytC levels, both remained elevated [99].

### 2.5. p38 MAPK, and Other Signaling Molecules

Two peaks of p38 MAPK were observed in KCs cultivated with PV-IgG and in a mouse model injected with PF-IgG. The first one occurred before the acantholysis, 30 min after addition of PV-IgG, and 2–4 h after PF-IgG injection in KCs and a mouse model, respectively. In KCs, cleaved PARP and positive TUNEL increased after 6 h of PV-IgG addition after the second peak of p38 MAPK. In the mouse model, the second peak of p38 MAPK occurred between 8–21 h after PF IgG injection and positive TUNEL, cleaved CASP-3 and cleaved PARP increased after acantholysis triggered by PF IgG injection. Interestingly, inhibition of the first peak of p38 MAPK blocked blistering. The inhibition of the p38 MAPK second peak did not block the blister formation. However, it blocked the activation of CASP-3 in mice that received the p38 MAPK inhibitor 4 h after injection of PF IgG [17] (Supplementary Table S5). Furthermore, inhibitors of executioner caspases (CASP-3 and 7) did not change EGFR, SRC, and the first peak of p38 MAPK, but decreased JNK, the second peak of p38 MAPK and CytC levels in keratinocytes treated with PV-IgG [99].

Mice treated with PV-IgG presented higher phosphorylation of the epidermal growth factor receptors HER1, HER2, HER3 and an upregulation of the HER ligands betacellulin, EGF, and transforming growth factor TGF-α as well as an increased expression of Src and mTOR. TUNEL positive cells increased, before blistering, MTOR mediates the apoptotic process and Akt promotes cell survival through regulation of pro-apoptotic molecules. PV-IgG injection up-regulated mTOR but down-regulated Akt, which might drive cells to apoptosis. In agreement, inhibition of HER, mTOR, pan-caspase, or Src decreases the number of apoptotic cells and acantholysis in PV neonatal mice. Inhibition of Src or HER resulted in a decrease of mTOR levels as well. Inhibition of HER resulted in P-HER and Src decreased [106].

The importance of focal adhesion kinase (FAK) in blister formation was shown by their absence in mice pretreated with FAK-inhibitor, before PV-IgG injection. Furthermore, the inhibition of mTOR, Src, and HER isoforms before the treatment with PV-IgG decreased the levels of phosphorylated FAK. Higher Bcl-2 levels and lower levels of Bax, CASP-3, and CASP-9 followed FAK inhibition compared to the injection of PV-IgG without any pretreatment [90] (Supplementary Table S5). 

## 3. Conclusions and Perspectives

In conclusion, we point to some possibilities to explain the divergent results for apoptosis as an upstream or downstream event of acantholysis: (1) many investigations were only descriptive, without enough statistical power for a meaningful analysis; (2) several of them limited their findings to the detection a specific molecule and/or TUNEL positive cells, frequently without a control group, neither with positive and negative controls for immunohistochemistry/ immunofluorescence [19,62,63,65,89,97,105,120,121,122]; (3) there is no follow-up investigation to ensure that acantholysis follows apoptosis in nonlesional areas presenting pro-apoptotic molecules, a common problem for human biopsies [80,107,120]; (4) the usage of only one or two IgG pools from pemphigus patients on cell lines and mouse models may bias the results due to the heterogeneity of their composition, including different anti-mitochondrial IgG, not purified for anti-DSG1/DSG3, which may trigger divergent cellular signaling and pro-apoptotic and/or pro-necrotic (oncotic) outcomes [6,24]; (5) PV serum was seldom incorporated in cell death investigations, but different outcomes result from the usage of PV-IgG or PV sera, revealing the importance of its components [94]; (6) the genetic regulation of cell death pathways was poorly investigated in pemphigus, despite the fact that patient genetic background may unveil the susceptibility to deregulated mechanisms that precede the production of anti-DSG1 and/or 3 IgG, before self-tolerance is broken. (7) most authors investigated only apoptosis, one of 12 cell death pathways, but the importance of other key cell death molecules and pathways, as those responsive to environmental factors for PF, cannot be underestimated; (8) a complex combination of genetic susceptibility background, pemphigus-IgG production and environmental factors as mosquito bites and calcium sequestrant components may ultimately trigger cell death in the disease, which must be controlled for and thoroughly investigated [37], (9) Lastly, multiple pleiotropic effects of the investigated molecules are important to account for (Supplementary Figure S2). Taken together, this review is intended to motivate future well-controlled, statistically relevant investigations on pemphigus and keratinocyte cell death pathways besides apoptosis, given the importance of this knowledge to overcome the urgent challenges of preventive, personalized, participative, and predictive Medicine for these autoimmune diseases. 

## Figures and Tables

**Table 1:** Studies included in this review-investigating cell death molecules in pemphigus.

References	Target(s) Investigated	Study Design			
		Patients	Human Skin Organ Culture	Cell Lines	Mouse Model
[73]	*TNF*	✓			
[74]	TNF	✓			
[24]	CASP3 *, CASP8 *, FASL *, FLIP1 *, CAPN1, DNA fragmentation, membrane cell permeability			✓	✓
[75]	TNF	✓			
[76]	iNOS *, TP53 *, *BCL2*, DNA fragmentation			✓	
[37]	*TNF*, *TRAF2*, *CD36*, *SIRPA*, *CD47*, *HK1*, *PAK2*, *EIF2AK3*, *RAPGEF3*, *PRKN*	✓			
[77]	CYCS			✓	
[78]	CYCS			✓	
[79]	CASP9, CYCS, SRC, p38 MAPK			✓	
[80]	CASP3, TNF, DNA fragmentation	✓			
[81]	TNF	✓			
[82]	DNA fragmentation	✓			
[83]	TNF	✓			
[84]	BAX, DNA fragmentation	✓			
[85]	*BAG4, BCL2L11, CSF2RA, CSF2RB, GNAQ, IL15, LEPROT, MAP3K1, MAP3K4, MAP3K5, MAPK9, NSMAF, PPP2CA, SMAD2, SMAD4, ERAP1, ERBB4, BDNF, IRAK1, TNFSF13*	✓			
[86]	*TNF*	✓			
[87]	TNF			✓	
[88]	TNF *	✓		✓	✓
[89]	CASP3, CASP8, membrane cell permeability, degraded cells and condensed chromatin	✓	✓	✓	
[19]	CASP3, CASP8, EGFR, ERK1/2, FASL, JUN, membrane cell permeability	✓	✓	✓	
[90]	BAX, BCL2, CASP3, CASP9, EGFR, MTOR, SRC, FAK				✓
[62]	DNA fragmentation	✓			
[63]	CASP3, CASP8, Fractin, PARP, DNA fragmentation	✓	✓		
[91]	*TNF*	✓			
[22]	mitochondrial damage			✓	
[92]	TNF	✓			
[93]	*TP53*, *BAX*	✓			
[94]	EIF2α, PERK			✓	
[17]	CASP3, p38 MAPK, PARP, DNA fragmentation			✓	✓
[95]	BAX, BCLXL, CASP3, CASP6, DNA fragmentation				✓
[96]	*BAX, BCL2*, *CASP3*, NOD2, RIPK2, NF-kB p-p65, ROS, membrane cell permeability			✓	
[97]	*TNF*	✓			
[61]	CASP8, FAS, FASL, DNA fragmentation	✓		✓	✓
[68]	CASP3, PARP, DNA fragmentation	✓		✓	✓
[98]	*ANXA4*, *BCL2A1*, *COP*, *GZMA*, *GZMH*, *LGALS1*, *LGALS12*, *NALP12*, *P2RX1, PIK3R1, PRKCA, SIAH1, TLR2, TNFRSF10A, TNFRSF18, TNFSF9, TNFSF10, TNFSF13*	✓			
[99]	CASP3, CASP8, CASP9, CYCS, EGFR, FASL, JNK, p38 MAPK, SRC			✓	
[100]	FASL	✓			
[101]	*TNF*	✓			
[102]	TNF	✓			
[103]	*BAX*, *CCND2*, *GADD34*, *PEA15*, *TSSC3*, *WAF1*			✓	
[104]	TNF, FASL, membrane cell permeability			✓	
[105]	CASP3 *, FAZ *, FASL *, DNA fragmentation	✓			
[106]	AKT, BTC, EGF, EGFR, HER2, HER3, P-SCR, TNF, MTOR, DNA fragmentation				✓
[107]	FASL, CASP8, DNA fragmentation	✓		✓	
[108]	TNF	✓			
[109]	BAX, CASP3, TP53	✓		✓	
[110]	IL1B			✓	
[65]	BCL2, FAS, IFNG, IL1-B, iNOS, TNF, DNA fragmentation	✓			
[111]	*TNF*	✓			
[112]	ST18	✓			
[113]	FAS	✓			
[64]	CASP3, FLIP, DNA fragmentation	✓		✓	
[114]	membrane cell permeability			✓	
[115]	*NLRP3*	✓			
[116]	membrane cell permeability		✓		
[117]	CXCL10, IFNG, TNF	✓			
[118]	LIVIN, XIAP	✓			
[119]	*TNF*, DNA fragmentation	✓			
[120]	ANXA5, BAX, BCL2, CASP1, CASP3, CASP8, FAS, FASL, FASR, TP53, DNA fragmentation, membrane cell permeability	✓	✓	✓	
[121]	BCL2, CASP1, CASP3, CASP8, FASL, TP53,			✓	
[122]	DNA fragmentation	✓			

* molecule investigated both at the gene and protein level. ✓investigated in… (see header). AKT, AKT serine/threonine kinase 1; *ANXA4*, annexin A4; ANXA5, annexin A5; *BAG4*, BAG cochaperone 4; BAX, BCL2 associated X apoptosis regulator; BCL2, BCL2 apoptosis regulator; *BCL2A1*, BCL2 related protein A1; *BCL2L11*, BCL2 like 11; *BDNF*, brain derived neurotrophic factor; BCLXL, BCL2-like 1; BTC, betacellulin; CAPN1, calpain 1; CASP1, caspase1; CASP3, caspase 3; CASP6, caspase 6; CASP7, caspase 7; CASP8, caspase 8; CASP9, caspase 9; *CCND2*, cyclin D2; CD36, CD36 molecule; CD47, CD47 molecule; *COP*, caspase recruitment domain-containing protein 16; CSF2RA, colony stimulating factor 2 receptor subunit alpha; *CSF2RB*, colony stimulating factor 2 receptor subunit beta; CXCL10, C-X-C motif chemokine ligand 10; CYCS, cytochrome c; DSC3, desmocollin 3; EGF, epidermal growth factor; EGFR, epidermal growth factor receptor; EIF2α, eukaryotic translation initiation factor 2 subunit alpha; EIF2AK3, eukaryotic translation initiation factor 2 alpha kinase 3; *ERAP1*, endoplasmic reticulum aminopeptidase 1; *ERBB4*, erb-b2 receptor tyrosine kinase 4; ERK1/2, mitogen-activated protein kinase; FAK, focal adhesion kinase; FAS, Fas cell surface death receptor; FASL, Fas ligand; FLIP1, TNFAIP interacting protein 2; FLIPL, CASP8 and FADD like apoptosis regulator; FLIPS; *GADD34*, protein phosphatase 1 regulatory subunit 15A; *GNAQ*, G protein subunit alpha q; *GZMA*, granzyme A; *GZMH*, granzyme H; HER, human epidermal growth factor receptor related; HER2, erb-b2 receptor tyrosine kinase 2; HER3, erb-b2 receptor tyrosine kinase 3; HK1, hexokinase 1; IFNG, interferon gamma; IL1, interleukin 1; IL1B, interleukin 1 beta; *IL15*, interleukin 15; iNOS, inducible nitric oxide synthase; *IRAK1*, interleukin 1 receptor associated kinase 1; JNK, c-Jun NH2-terminal kinase; JUN, AP-1 transcription factor subunit; *LEPROT*, leptin receptor overlapping transcript; *LGALS1*, galectin 1; *LGALS12*, galectin 12; LIVIN, baculoviral IAP repeat containing 7; M3AR, M3 muscarinic acetylcholine receptor; *MAP3K1*, mitogen-activated protein kinase kinase kinase 1; *MAP3K4*, mitogen-activated protein kinase kinase kinase 4; *MAP3K5*, mitogen-activated protein kinase kinase kinase 5; *MAPK9*, mitogen-activated protein kinase kinase kinase 9; MTOR, mechanistic target of rapamycin kinase; *NALP12*, NLR family pyrin domain containing 12; NF-kB p-p65, nuclear factor kappa B p-p65; *NLRP3*, NLR family pyrin domain containing 3; NOD2, nucleotide binding oligomerization domain containing 2; *NSMAF*, neutral sphingomyelinase activation associated factor; *P2RX1*, purinergic receptor P2X 1; p38 MAPK, p38 mitogen-activated protein kinase; PAK2, p21 (RAC1) activated kinase 2; PARP, poly-(ADP-ribose) polymerase; *PEA15*, proliferation and apoptosis adaptor protein 15; PERK; *PIK3R1*, phosphoinositide-3-kinase regulatory subunit 1; *PPP2CA*, protein phosphatase 2 catalytic subunit alpha; *PRKCA*, protein kinase C alpha; PRKN, parkin RBR E3 ubiquitin protein ligase; RAPGEF3, Rap guanine nucleotide exchange factor 3; RIPK2, receptor interacting serine/threonine kinase 2; ROS, reactive oxygen species; *SIAH1*, siah E3 ubiquitin protein ligase 1; SIRPA, signal regulatory protein alpha; *SMAD2*, SMAD family member 2; *SMAD4*, SMAD family member 4; SPCA1, secretory pathway Ca^2+^/ Mn^2+^-ATPase isoform 1; SRC, proto-oncogene tyrosine-protein kinase Src; ST18, ST18 C2H2C-type zinc finger transcription factor; *TLR2*, toll-like receptor 2; TNF, tumor necrosis factor; *TNFRSF10A*, TNF receptor superfamily member 10a; *TNFRSF18*, TNF receptor superfamily member 18; *TNFSF9*, TNF superfamily member 9; *TNFSF10*, TNF superfamily member 10; *TNFSF13*, TNF superfamily member 13; TP53, tumor protein p53; TRAF2, TNF receptor associated factor 2; *TSSC3*, pleckstrin homology like domain family A member 2; XIAP, X-linked inhibitor of apoptosis; *WAF1*, cyclin dependent kinase inhibitor 1A.

**Table 2:** Reported alterations in protein levels after blister formation in pemphigus patients.

Protein	Detected or Increased			Absent, Low or Decreased		
	PV	PF	PV and PF	PV	PF	PV and PF
BAX	[120] *			[84]		
BCL2		[65] *		[120] *		
CASP-1	[120] *					
CASP-3	[105] * [109] [80] [120] *			[64] [89] *		[63] *
CASP-8	[120] * [89] *					[63] *
CXCL-10				[117]		
EGFR	[19] *					
FAS	[105] * [61] [113] [120] *	[65] *				
FAS-L	[100] * [105] * [120] *		[107]			[107] +
Fractin						[63] *
IFN-G	[117]	[65] *				
IL-1B		[65] *				
iNOS		[65] *				
LIVIN	[118] +					
ST18	[112]					
PARP						[63] *
TNF	[92] [83] [74] [108] [80] [75] #	[65] *	[81]	[75] #,+ [117] [102] [83] +		
TP53	[109] [120] *					
XIAP	[118] +					

BAX, BCL2 associated X apoptosis regulator; BCL2, BCL2 apoptosis regulator; CASP1, caspase1; CASP3, caspase 3; CASP8, caspase 8; CXCL10, C-X-C motif chemokine ligand 10; FAS, Fas cell surface death receptor; FASL, Fas ligand; IFNG, interferon-gamma; iNOS, inducible nitric oxide synthase; LIVIN, baculoviral IAP repeat-containing 7; ST18, ST18 C2H2C-type zinc finger transcription factor; PARP, poly-(ADP-ribose) polymerase; TNF, tumor necrosis factor; TP53, tumor protein p53; XIAP, X-linked inhibitor of apoptosis; PF, pemphigus foliaceus; PV, pemphigus vulgaris. * Statistical significance was not mentioned in the article. + After therapeutic intervention. # Case report.

**Table 3:** Reported alterations in DNA degradation by Terminal Deoxynucleotidyl Transferase-Mediated dUTP Nick end Labeling Assay (TUNEL) or in cell membrane integrity by Trypan Blue or Annexin 5.

Type	Time	Detected or Increased			Absent, without Difference		
		PV	PF	PV and PF	PV	PF	PV and PF
HUMANS	After blister formation in patients	[105] * [120] * [80] [84] [82]	[65] *	[107] [62]*	[68] [64]	[122] *	[63] *
CELL LINES	After treatment with PF/PV IgG (before keratinocytes dissociation)	[120] * [17]			[68]		
	After keratinocytes dissociation	[76] [24] [120] * [104] [104] + [89] * [19] * [19] +,* [121] * [96] [114]		[107]	[68] [64]		
	After some therapeutic intervention in cell lines			[17] [107]	[19] * [24] [120] * [96]		[107]
MOUSE MODELS	After injection with PF/PV IgG (before blistering formation)	[61] [106]	[95]		[68]		
	After blistering formation	[24]	[17]		[68]		
	After some therapeutic intervention				[24] [106]	[95]	

PF, pemphigus foliaceus; PV, pemphigus vulgaris; IgG, immunoglobulin G. +: tissue culture or human skin organ culture. * Statistical significance was not mentioned in the article.

## Data Availability

Not applicable.

## Supplementary Materials

The following supporting information can be downloaded at: https://www.mdpi.com/article/10.3390/life12030329/s1, Figure S1: PRISMA study selection diagram flow [141,142]; Figure S2: Wikipathways of molecules involved in cell death pathways. Table S1: Genetic background in pemphigus vulgaris and pemphigus foliaceus patients; Table S2: Reported alterations in mRNA expression after blister formation in patients; Table S3: Reported alterations in mRNA expression after keratinocytes dissociation in cell lines; Table S4: Reported alterations in protein levels in cell lines; Table S5: Reported alterations in protein levels in a mouse model.
