# Supplementary material for: Marked to Die-Cell Death Mechanisms for Keratinocyte Acantholysis in Pemphigus Diseases
Source: Life (Basel). 2022 Feb 22;12(3):329. doi: 10.3390/life12030329 (PMC8948972; doi:10.3390/life12030329)
Supplement: Supplementary file 1 [file life-12-00329-s001.zip › life-1593205-Supplementary Materials.pdf]

life

MDPI

SUPPLEMENTARY MATERIAL

Market to Die-Cell Death Mechanisms for Keratinocyte Acantholysis in Pemphigus Diseases

1. Materials and Methods

To investigate the relationships between cell death pathways and pemphigus, a PubMed literature search was performed. Articles according to the criteria listed below and published until April 20, 2021, were included.

1.1. Search strategy

All original research and review articles were searched in the electronic database PubMed using the term ("pemphigus"[MeshTerm]) combined, through the descriptor "AND", with ("cell death"[MeshTerm]), ("apoptosis"[MeshTerm]), ("necrosis"[MeshTerm]), ("necroptosis"[MeshTerm]), ("ferroptosis"[MeshTerm]), ("pyroptosis"[MeshTerm]), ("parthanatos"[MeshTerm]), ("entotic"[MeshTerm]), ("NETotic"[MeshTerm]), ("lysosome-dependent"[MeshTerm]), ("autophagy-dependent"[MeshTerm]), and ("immunogenic cell death"[MeshTerm]). Articles not related to the human species were excluded. 251 articles were selected, 32 being duplicates. Thus, 219 were kept for analysis.

1.2. Inclusion criteria

All studies that met the following criteria were included: (1) the article refers to pemphigus vulgaris or foliaceus; and (2) address cell death pathways in these diseases. Exclusion criteria for language were not applied. After these steps, 84 articles were determined for full reading and final check-up. 61 articles were included in the review [141] (**Supp. Figure 1**).

1.3. Data extraction

The selection process was carried out using Rayyan software [142] by two researchers. Data extraction was performed by four independent reviewers and, in parallel, checked by another. All relevant information was classified in a spreadsheet, such as the molecules investigated in each study, variations in their expression, the chronology of interventions, as well as their respective results, in addition to the method by which they were obtained and the means (e.g. human, animal or cell lines).

Supplementary Figure S1. PRISMA study selection diagram flow.

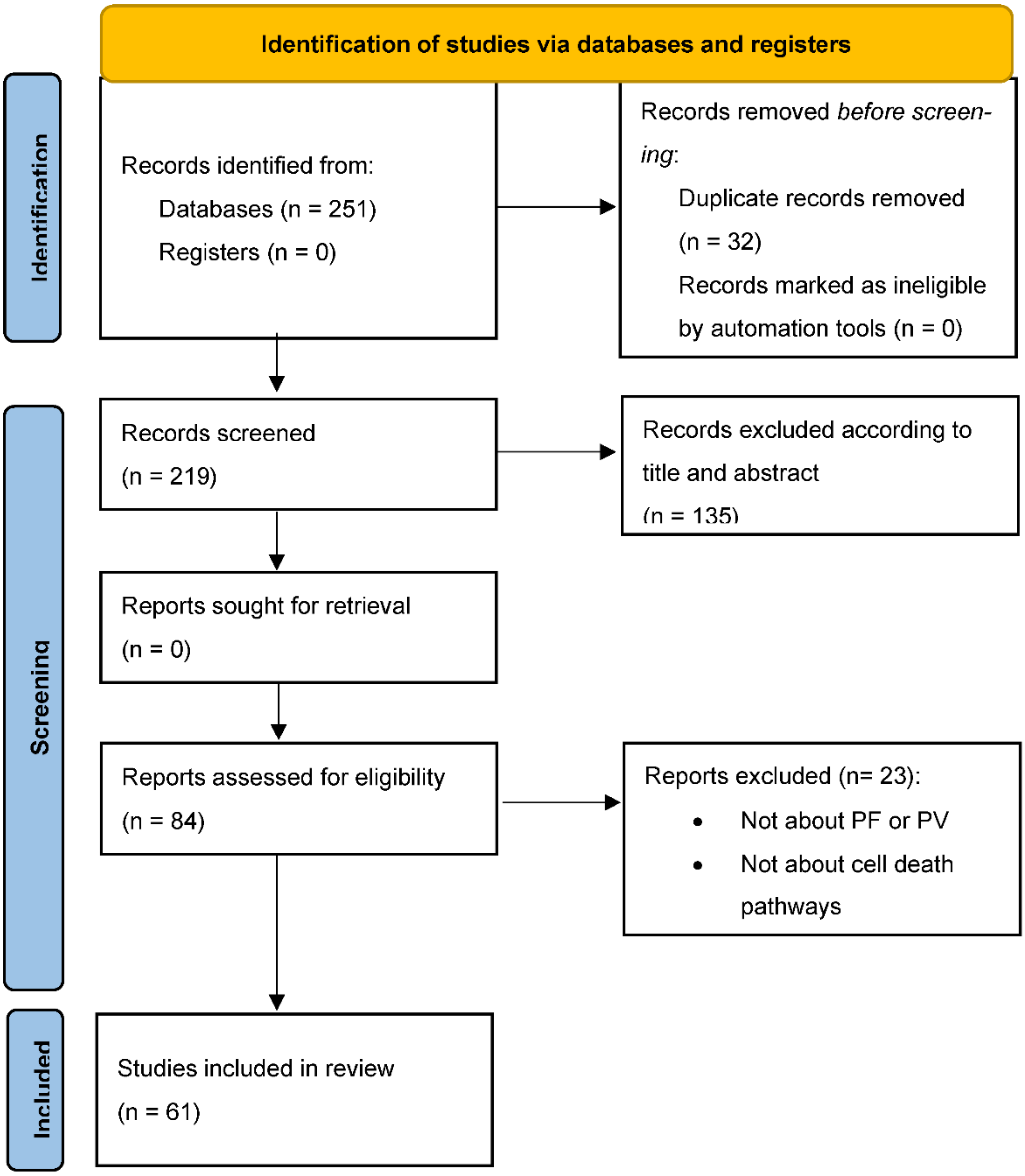

2. Supplementary tables

Supplementary Table S1. Genetic background in pemphigus vulgaris and pemphigus foliaceus patients.

Gene	Genetic variant	Associated		Not Associated	
		PV	PF	PV	PF
BAX	*rs4645878 G>A (−248)*				[93]
CD36	*rs4112274*T*		[37]		
CD47	*rs12695175*G*		[37]		
EIF2AK3	*rs10167879*T*		[37]		
HK1	*rs7072268*T*		[37]		
PAK2	*rs9325377*A*		[37]		
PRKN	*rs9355950*C*		[37]		
RAPGEF3	*rs10747521*A*		[37]		
SIRPA	*rs6075340*A*		[37]		
ST18	*rs2304365*A/A*	[112]			
ST18	*rs4074067*T*	[112]			
ST18	*rs10504140*A*	[112]			
TNF	*rs1800630*A (-863)*		[37]		[111]
TNF	*rs1800629*A (308*A)*		[119]	[101]<br>[86]<br>[91]	[101]<br>[111]<br>[86]
TNF	*rs1800629*A/rs361525*G (308*G/238*G)*	[91]			
TNF	*rs1800629 GA+AA (308)*	[101]			[101]
TNF	*rs361525*A (238*A)*		[111]		
TNF	*rs1800629*G / rs361525*A (308*G/238*A)*		[111]		
TNF	*STR *2, *5, *6*		[73]		
TNF	*STR *a2 / *a5*		[73]		
TP53	*rs1042522 C>G (12139)*				[93]
TRAF2	*rs10781522*G*		[37]		

[73]: Tunisian EPF, [37]: Brazilian EPF, [93]: Brazilian EPF, [86]: Argentinian population. STR: microsatellite. *BAX*, BCL2 associated X apoptosis regulator; *CD36*, CD36 molecule; *CD47*, CD47 molecule; *EIF2AK3*, eukaryotic translation initiation factor 2 alpha kinase 3; *HK1*, hexokinase 1; *PAK2*, p21 (RAC1) activated kinase 2; *PRKN*, parkin RBR E3 ubiquitin protein ligase; *RAPGEF3*, Rap guanine nucleotide exchange factor 3; *SIRPA*, signal regulatory protein alpha; *ST18*, ST18 C2H2C-type zinc finger transcription factor; *TNF*, tumor necrosis factor; *TP53*, tumor protein p53; *TRAF2*, TNF receptor associated factor 2; PF, pemphigus foliaceus; PV, pemphigus vulgaris.

Supplementary Table S2. Reported alterations in mRNA expression after blister formation in patients.

mRNA	Increased		Decreased	
	PV	PF	PV	PF
ANXA4		[98]		
BAG4	[85]			
BCL2A1		[98]+		
BCL2L11	[85]			
BDNF			[85]	
CASP3	[105]*			
COP		[98]		
CSF2RA	[85]			
CSF2RB	[85]			
ERAP1	[85]			
ERBB4			[85]	
FAS	[105]*			
FASL	[105]*			
GNAQ	[85]			
GZMA		[98]		
GZMH		[98]		
IL15	[85]			
IRAK1	[85]			
LEPROT	[85]			
LGALS1		[98]		
		[98]+		
LGALS12		[98]		
		[98]+		
MAP3K1	[85]			
MAP3K4	[85]			
MAP3K5	[85]			
MAPK9	[85]			
NALP12		[98]		
NLRP3				
NSMAF	[85]			
P2RX1		[98]+		
PIK3R1				[98]+
PPP2CA	[85]			
PRKCA				[98]
SIAH1				[98]
SMAD2	[85]			
SMAD4	[85]			
TLR2		[98]		
TNF	[88]	[97]*		

	[97]*		
TNFRSF10A			[98]+
TNFRFS18		[98]	
TNFSF9		[98]	
TNFSF10		[98]	
TNFSF13	[85]		
TNFSF13B		[98]	
		[98]+	

ANXA4, annexin A4; *BAG4*, BAG cochaperone 4; *BCL2A1*, BCL2 related protein A1; *BCL2L11*, BCL2 like 11; *BDNF*, brain derived neurotrophic factor; *CASP3*, caspase 3; *COP*, caspase recruitment domain-containing protein 16; *CSF2RA*, colony stimulating factor 2 receptor subunit alpha; *CSF2RB*, colony stimulating factor 2 receptor subunit beta; *ERAP1*, endoplasmic reticulum aminopeptidase 1; *ERBB4*, erb-b2 receptor tyrosine kinase 4; *FAS*, Fas cell surface death receptor; *FASL*, Fas ligand; *GNAQ*, G protein subunit alpha q; *GZMA*, granzyme A; *GZMH*, granzyme H; *IL15*, interleukin 15; *IRAK1*, interleukin 1 receptor associated kinase 1; *LEPROT*, leptin receptor overlapping transcript; *LGALS1*, galectin 1; *LGALS12*, galectin 12; *MAP3K1*, mitogen-activated protein kinase kinase kinase 1; *MAP3K4*, mitogen-activated protein kinase kinase kinase 4; *MAP3K5*, mitogen-activated protein kinase kinase kinase 5; *MAPK9*, mitogen-activated protein kinase kinase kinase 9; *NALP12*, NLR family pyrin domain containing 12; *NLRP3*, NLR family pyrin domain containing 3; *NSMAF*, neutral sphingomyelinase activation associated factor; *P2RX1*, purinergic receptor P2X 1; *PIK3R1*, phosphoinositide-3-kinase regulatory subunit 1; *PPP2CA*, protein phosphatase 2 catalytic subunit alpha; *PRKCA*, protein kinase C alpha; *SIAH1*, siah E3 ubiquitin protein ligase 1; *SMAD2*, SMAD family member 2; *SMAD4*, SMAD family member 4; *TLR2*, toll-like receptor 2; *TNF*, tumor necrosis factor; *TNFRSF10A*, TNF receptor superfamily member 10a; *TNFRSF18*, TNF receptor superfamily member 18; *TNFSF9*, TNF superfamily member 9; *TNFSF10*, TNF superfamily member 10; *TNFSF13*, TNF superfamily member 13. PF, pemphigus foliaceus; PV, pemphigus vulgaris. +After therapeutic intervention. *Statistical significance was not mentioned in the article.

Supplementary Table S3. Reported alterations in mRNA expression after keratinocytes dissociation in cell lines.

mRNA	Increased		Decreased or without difference	
	**IgG-PV**	**After therapeutic intervention**	**IgG-PV**	**After therapeutic intervention**
BAX	[96]	[103]		[96]
BCL2			[96]<br>[76]	
CASP3	[96]<br>[24]			[24]<br>[96]
CASP8	[24]			[24]
CCND2			[103]	
FASL	[24]			[24]
FLIP1	[24]	[24]		
GADD34			[103]	
iNOS	[76]			
PEA15			[103]	
TNF	[88]#			
TP53	[76]			
TSSC3			[103]	
WAF1			[103]	

BAX, BCL2 associated X apoptosis regulator; *BCL2*, BCL2 apoptosis regulator; *CASP3*, caspase 3; *CASP8*, caspase 8; *CCND2*, cyclin D2; *FASL*, Fas ligand; *FLIP1*, TNFAIP interacting protein 2; *GADD34*, protein phosphatase 1 regulatory subunit 15A; *iNOS*, inducible nitric oxide synthase; *PEA15*, proliferation and apoptosis adaptor protein 15; *TNF*, tumor necrosis factor; *TP53*, tumor protein p53; *TSSC3*, pleckstrin homology like domain family A member 2; *WAF1*, cyclin dependent kinase inhibitor 1A. PF, pemphigus foliaceus; PV, pemphigus vulgaris. #Before keratinocytes dissociation. *Statistical significance was not mentioned in the article.

Supplementary Table S4. Reported alterations in protein levels in cell lines.

Protein	Detected or increased		Absent, low or decreased	
	PV	After therapeutic intervention	PV	After therapeutic intervention
ANXA-5	[120]*#			
BAX	[109]			
BCL2		[96]	[120]*<br>[121]*	
CAPN-1	[24]			
CASP-1	[120]*#<br>[121]*			
CASP-3	[120]*#<br>[121]*<br>[24]<br>[89]*<br>[19]*<br>[99]#		[68]#<br>[68]	[19]*<br>[99]
CASP-8	[120]*<br>[121]*<br>[24]<br>[89]*#<br>[99]#			[61]<br>[99]
CASP-9	[99]#<br>[99]<br>[79]			[99]
c-Jun	[19]*#<br>[99]#			[19]*
CYCS	[99]<br>[79]			[79]<br>[99]
EGFR	[77]#<br>[19]*#<br>[99]#			
p-ERK1/2	[19]*#			[19]*
FAS	[120]*#			
FASL	[120]*<br>[120]*#<br>[121]*#<br>[24]			[19]*
FLIP1	[24]			
IL1B		[110]		
iNOS	[76]			
JNK	[99]#			[99]
NF-kB p-p65	[96]			[96]
NOD2	[96]			[96]
RIPK2	[96]			[96]
SRC	[99]#<br>[79]			
p38 MAPK	[99]#<br>[99]<br>[17]#<br>[79]			[99]

PARP	[17]#	[68]
p-EIF2α	[94]#	
p-PERK	[94]#	
TNF	[120]*	
	[76]	
TP53	[109]	
	[120]*	

ANXA5, annexin A5; BAX, BCL2 associated X apoptosis regulator; BCL2, BCL2 apoptosis regulator; CAPN1, calpain 1; CASP1, caspase1; CASP3, caspase 3; CASP8, caspase 8; CASP9, caspase 9; CYCS, cytochrome c; EGFR, epidermal growth factor receptor; ERK1/2, mitogen-activated protein kinase; FAS, Fas cell surface death receptor; FASL, Fas ligand; FLIP1, TNFAIP interacting protein 2; GADD34, protein phosphatase 1 regulatory subunit 15A; IL1B, interleukin 1 beta; iNOS, inducible nitric oxide synthase; JNK, c-Jun NH2-terminal kinase; JUN, AP-1 transcription factor subunit; NF-kB p-p65, nuclear factor kappa B p-p65; NOD2, nucleotide binding oligomerization domain containing 2; RIPK2, receptor interacting serine/threonine kinase 2; SRC, proto-oncogene tyrosine-protein kinase Src; p38MAPK, p38 mitogen-activated protein kinase; PARP, poly-(ADP-ribose) polymerase; p-EIF2α, eukaryotic translation initiation factor 2 subunit alpha; PERK, eukaryotic translation initiation factor 2 alpha kinase 3; TNF, tumor necrosis factor; TP53, tumor protein p53. PF, pemphigus foliaceus; PV, pemphigus vulgaris. #Before keratinocytes dissociation. *Statistical significance was not mentioned in the article.

Supplementary Table S5. Reported alterations in protein levels in mouse model.

Protein	Detected or increased		Absent, low or decreased	
	PV	PF	PV	PF
AKT			[106]	
BAX	[90]	[95]#	[90]+	
BCL2	[90]+		[90]	
BCL-XL				[95]#<br>[95]
BTC	[106]			
CASP3	[90]	[95]#<br>[17]	[68]#<br>[68]<br>[90]+	[17]+
CASP6		[95]#		
CASP9	[90]		[90]+	
CYCS	[77]			
EGF	[106]		[106]+	
EGFR	[106]			
FAK			[90]+	
FASL	[61]#			
HER2	[106]			
HER3	[106]			
MTOR	[106]		[106]+	
p38 MAPK		[17]#<br>[17]		
PARP		[17]	[68]	
SRC	[106]		[106]+	
TNF	[106]			

AKT, AKT serine/threonine kinase 1; BAX, BCL2 associated X apoptosis regulator; BCL2, BCL2 apoptosis regulator; BCLXL, BCL2-like 1; BTC, betacellulin; CASP3, caspase 3; CASP6, caspase 6; CASP9, caspase 9; CYCS, cytochrome c; EGF, epidermal growth factor; EGFR, epidermal growth factor receptor; FAK, focal adhesion kinase; FASL, Fas ligand; HER2, erb-b2 receptor tyrosine kinase 2; HER3, erb-b2 receptor tyrosine kinase 3; MTOR, mechanistic target of rapamycin kinase; p38MAPK, p38 mitogen-activated protein kinae; PARP, poly-(ADP-ribose) polymerase; SRC, proto-oncogene tyrosine-protein kinase Src; TNF, tumor necrosis factor; PF, pemphigus foliaceus; PV, pemphigus vulgaris. +After therapeutic intervention. #Before blistering formation. *Statistical significance was not mentioned in the article.

Supplementary Figure S2. Wikipathways of molecules involved in cell death pathways. Representative for the pleiotropic effect of the investigated molecules. Search: https://fuma.ctglab.nl/.

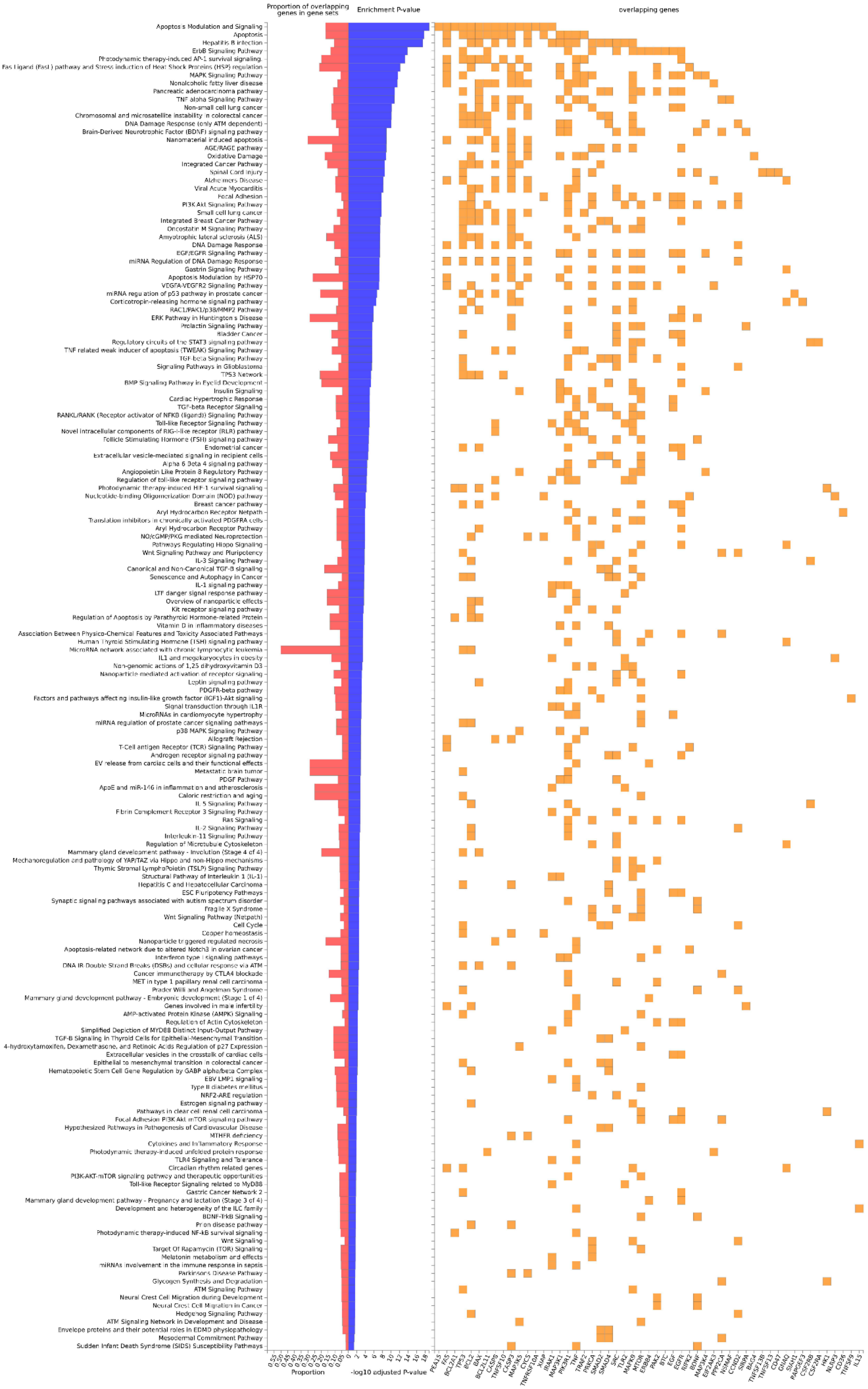
Proportion of overlapping genes in gene sets
Enrichment P-value
overlapping genes
Apoptosis Modulation and Signaling
Apoptosis
Hepatitis B infection
ErbB Signaling Pathway
Photodynamic therapy-induced AP-1 survival signaling.
Fas Ligand (FasL) pathway and Stress induction of Heat Shock Proteins (HSP) regulation
MAPK Signaling Pathway
Nonalcoholic fatty liver disease
Pancreatic adenocarcinoma pathway
TNF alpha Signaling Pathway
Non-small cell lung cancer
Chromosomal and microsatellite instability in colorectal cancer
DNA Damage Response (only ATM dependent)
Brain-Derived Neurotrophic Factor (BDNF) signaling pathway
Nanomaterial induced apoptosis
AGE/RAGE pathway
Oxidative Damage
Integrated Cancer Pathway
Spinal Cord Injury
Alzheimers Disease
Viral Acute Myocarditis
Focal Adhesion
PI3K-Akt Signaling Pathway
Small cell lung cancer
Integrated Breast Cancer Pathway
Oncostatin M Signaling Pathway
Amyotrophic lateral sclerosis (ALS)
DNA Damage Response
EGF/EGFR Signaling Pathway
miRNA Regulation of DNA Damage Response
Gastrin Signaling Pathway
Apoptosis Modulation by HSP70
VEGFA-VEGFR2 Signaling Pathway
miRNA regulation of p53 pathway in prostate cancer
Corticotropin-releasing hormone signaling pathway
RAC1/PAK1/p38/MMP2 Pathway
ERK Pathway in Huntington's Disease
Prolactin Signaling Pathway
Bladder Cancer
Regulatory circuits of the STAT3 signaling pathway
TNF related weak inducer of apoptosis (TWEAK) Signaling Pathway
TGF-beta Signaling Pathway
Signaling Pathways in Glioblastoma
TP53 Network
BMP Signaling Pathway in Eyelid Development
Insulin Signaling
Cardiac Hypertrophic Response
TGF-beta Receptor Signaling
RANKL/RANK (Receptor activator of NFKB (ligand)) Signaling Pathway
Toll-like Receptor Signaling Pathway
Novel intracellular components of RIG-I-like receptor (RLR) pathway
Follicle Stimulating Hormone (FSH) signaling pathway
Endometrial cancer
Extracellular vesicle-mediated signaling in recipient cells
Alpha 6 Beta 4 signaling pathway
Angiopoietin Like Protein 8 Regulatory Pathway
Regulation of toll-like receptor signaling pathway
Photodynamic therapy-induced HIF-1 survival signaling
Nucleotide-binding Oligomerization Domain (NOD) pathway
Breast cancer pathway
Aryl Hydrocarbon Receptor Netpath
Translation inhibitors in chronically activated PDGFRA cells
Aryl Hydrocarbon Receptor Pathway
NO/cGMP/PKG mediated Neuroprotection
Pathways Regulating Hippo Signaling
Wnt Signaling Pathway and Pluripotency
IL-3 Signaling Pathway
Canonical and Non-Canonical TGF-B signaling
Senescence and Autophagy in Cancer
IL-1 signaling pathway
LTF danger signal response pathway
Overview of nanoparticle effects
Kit receptor signaling pathway
Regulation of Apoptosis by Parathyroid Hormone-related Protein
Vitamin D in inflammatory diseases
Association Between Physico-Chemical Features and Toxicity Associated Pathways
Human Thyroid Stimulating Hormone (TSH) signaling pathway
MicroRNA network associated with chronic lymphocytic leukemia
IL1 and megakaryocytes in obesity
Non-genomic actions of 1,25 dihydroxyvitamin D3
Nanoparticle-mediated activation of receptor signaling
Leptin signaling pathway
PDGFR-beta pathway
Factors and pathways affecting insulin-like growth factor (IGF1)-Akt signaling
Signal transduction through IL1R
MicroRNAs in cardiomyocyte hypertrophy
miRNA regulation of prostate cancer signaling pathways
p38 MAPK Signaling Pathway
Allograft Rejection
T-Cell antigen Receptor (TCR) Signaling Pathway
Androgen receptor signaling pathway
EV release from cardiac cells and their functional effects
Metastatic brain tumor
PDGF Pathway
ApoE and miR-146 in inflammation and atherosclerosis
Caloric restriction and aging
IL-5 Signaling Pathway
Fibrin Complement Receptor 3 Signaling Pathway
Ras Signaling
IL-7 Signaling Pathway
Interleukin-11 Signaling Pathway
Regulation of Microtubule Cytoskeleton
Mammary gland development pathway - Involution (Stage 4 of 4)
Mechanoregulation and pathology of YAP/TAZ via Hippo and non-Hippo mechanisms
Thymic Stromal LymphoPoietin (TSLP) Signaling Pathway
Structural Pathway of Interleukin 1 (IL-1)
Hepatitis C and Hepatocellular Carcinoma
ESC Pluripotency Pathways
Synaptic signaling pathways associated with autism spectrum disorder
Fragile X Syndrome
Wnt Signaling Pathway (Netpath)
Cell Cycle
Copper homeostasis
Nanoparticle triggered regulated necrosis
Apoptosis-related network due to altered Notch3 in ovarian cancer
Interferon type I signaling pathways
DNA IR-Double Strand Breaks (DSBs) and cellular response via ATM
Cancer immunotherapy by CTLA4 blockade
MET in type 1 papillary renal cell carcinoma
Prader-Willi and Angelman Syndrome
Mammary gland development pathway - Embryonic development (Stage 1 of 4)
Genes involved in male infertility
AMP-activated Protein Kinase (AMPK) Signaling
Regulation of Actin Cytoskeleton
Simplified Depiction of MYD88 Distinct Input-Output Pathway
TGF-B Signaling in Thyroid Cells for Epithelial-Mesenchymal Transition
4-hydroxytamoxifen, Dexamethasone, and Retinoic Acids Regulation of p27 Expression
Extracellular vesicles in the crosstalk of cardiac cells
Epithelial to mesenchymal transition in colorectal cancer
Hematopoietic Stem Cell Gene Regulation by GABP alpha/beta Complex
EBV LMP1 signaling
Type II diabetes mellitus
NRF2-ARE regulation
Estrogen signaling pathway
Pathways in clear cell renal cell carcinoma
Focal Adhesion-PI3K-Akt-mTOR-signaling pathway
Hypothesized Pathways in Pathogenesis of Cardiovascular Disease
MTHFR deficiency
Cytokines and Inflammatory Response
Photodynamic therapy-induced unfolded protein response
TLR4 Signaling and Tolerance
Circadian rhythm related genes
PI3K-AKT-mTOR signaling pathway and therapeutic opportunities
Toll-like Receptor Signaling related to MyD88
Gastric Cancer Network 2
Mammary gland development pathway - Pregnancy and lactation (Stage 3 of 4)
Development and heterogeneity of the ILC family
BDNF-TrkB Signaling
Prion disease pathway
Photodynamic therapy-induced NF-kB survival signaling
Wnt Signaling
Target Of Rapamycin (TOR) Signaling
Melatonin metabolism and effects
miRNAs involvement in the immune response in sepsis
Parkinsons Disease Pathway
Glycogen Synthesis and Degradation
ATM Signaling Pathway
Neural Crest Cell Migration during Development
Neural Crest Cell Migration in Cancer
Hedgehog Signaling Pathway
ATM Signaling Network in Development and Disease
Envelope proteins and their potential roles in EDMD physiopathology
Mesodermal Commitment Pathway
Sudden Infant Death Syndrome (SIDS) Susceptibility Pathways
0.55 0.50 0.45 0.40 0.35 0.30 0.25 0.20 0.15 0.10 0.05 0
Proportion
0 2 4 6 8 10 12 14 16 18
-log10 adjusted P-value
PEA15 FAS BCL2L1 TP53 BCL2 BAX BCL2L11 CASP8 TNFSF10 CASP3 MAP3K5 CYCS TNFRSF10A XIAP IRAK1 MAP3K1 PIK3R1 TNF TRAF2 PRKCA SMAD2 SMAD4 SRC TLR2 MAPK8 MTOR ERBB4 PAK2 BTC EGF EGFR TIRAP BDNF MAP3K4 EIF2AK3 PPP2CA NOXA1 CCND1 SIRPA BAG4 TNFSF13B TNFSF13 CD47 GNAQ SIAH1 PARP2 CSF3R CSF2RB HK1 NLRP3 CD28 TNFSF9 IL15
